# Producing and Testing Prototype Tissue-Engineered 3D Tri-Leaflet Valved Stents on Biodegradable Poly-ε-Caprolactone Scaffolds

**DOI:** 10.3390/ijms242417357

**Published:** 2023-12-11

**Authors:** Georg Lutter, Nina Sophie Pommert, Xiling Zhang, Jette Seiler, Monireh Saeid Nia, David Meier, Stephanie L. Sellers, Stanislav N. Gorb, Jan-Hinnerk Hansen, Hatim Seoudy, Oliver J. Müller, Mohammed Saad, Assad Haneya, Derk Frank, Thomas Puehler, Janarthanan Sathananthan

**Affiliations:** 1 Department of Cardiac Surgery, University Hospital Schleswig-Holstein (UKSH), 24105 Kiel, Germany; ninasophie.pommert@uksh.de (N.S.P.); zhang_xiling@outlook.com (X.Z.); monireh.saeidnia@uksh.de (M.S.N.); assad.haneya@uksh.de (A.H.); thomas.puehler@uksh.de (T.P.); 2 DZHK (German Centre for Cardiovascular Research), Partner Site Hamburg/Kiel/Lübeck, 69120 Hamburg, Germany; janhinnerk.hansen@uksh.de (J.-H.H.); hatim.seoudy@uksh.de (H.S.); oliver.mueller@uksh.de (O.J.M.); mohammed.saad@uksh.de (M.S.); derk.frank@uksh.de (D.F.); 3 Department of Cardiology, Lausanne University Hospital and University of Lausanne, 1015 Lausanne, Switzerland; david.meier1291@gmail.com; 4 Centre for Cardiovascular Innovation, St Paul’s and Vancouver General Hospital, Vancouver, BC V6Z 1Y6, Canada; ssellers@providencehealth.bc.ca (S.L.S.); jsathananthan@providencehealth.bc.ca (J.S.); 5 Cardiovascular Translational Laboratory, Providence Research & Centre for Heart Lung Innovation, Vancouver, BC V6Z 1Y6, Canada; 6 Centre for Heart Valve Innovation, St. Paul’s Hospital, University of British Columbia, Vancouver, BC V6T 1Z4, Canada; 7 Department of Functional Morphology and Biomechanics, Zoological Institute, Christian-Albrecht University of Kiel, 24105 Kiel, Germany; 8 Department of Congenital Heart Disease and Pediatric Cardiology, University Hospital Schleswig-Holstein, 24105 Kiel, Germany; 9 Department of Cardiology and Angiology, University Hospital Schleswig-Holstein (UKSH), 24105 Kiel, Germany

**Keywords:** tissue engineering, heart valve, iMSCs, ECFCs, pMSCs, PCL nanofibers, biodegradable scaffold, biodegradable stent, transcatheter, transfemoral, endovascular, valve repair, valve replacement

## Abstract

Transcatheter pulmonary valve replacement is a minimally-invasive alternative treatment for right ventricular outflow tract dysfunction and has been rapidly evolving over the past years. Heart valve prostheses currently available still have major limitations. Therefore, one of the significant challenges for the future is the roll out of transcatheter tissue engineered pulmonary valve replacement to more patients. In the present study, biodegradable poly-ε-caprolactone (PCL) nanofiber scaffolds in the form of a 3D leaflet matrix were successfully seeded with human endothelial colony-forming cells (ECFCs), human induced pluripotent stem cell-derived MSCs (hMSCs), and porcine MSCs (pMSCs) for three weeks for the generation of 3D tissue-engineered tri-leaflet valved stent grafts. The cell adhesion, proliferation, and distribution of these 3D heart leaflets was analyzed using fluorescence microscopy and scanning electron microscopy (SEM). All cell lineages were able to increase the overgrown leaflet area within the three-week timeframe. While hMSCs showed a consistent growth rate over the course of three weeks, ECFSs showed almost no increase between days 7 and 14 until a growth spurt appeared between days 14 and 21. More than 90% of heart valve leaflets were covered with cells after the full three-week culturing cycle in nearly all leaflet areas, regardless of which cell type was used. This study shows that seeded biodegradable PCL nanofiber scaffolds incorporated in nitinol or biodegradable stents will offer a new therapeutic option in the future.

## 1. Introduction

Tissue engineering of heart valves aims to create functional, biocompatible, and durable replacements for damaged or diseased valves. Current approaches focus on utilizing living cells and biodegradable scaffolds to create a functional valve structure that mimics the mechanical and biological properties of native valves [1].

Scaffold design and material selection play a critical role in the success of tissue-engineered heart valves. The scaffold must provide mechanical support and allow for cell attachment and proliferation, all while withstanding physiological conditions. Additionally, the scaffold should be biocompatible and non-toxic. Biodegradable scaffolds can reduce the risk of long-term complications associated with non-degradable materials, such as inflammation, infection, or calcification. Despite important progress made in the development of tissue-engineered heart valves, no prototype has yet made it to the stage of routine clinical use.

Moreover, a major limitation of current valve prostheses is the absence of cells required for active repair and remodeling of their valve matrix. Leaflets of native human heart valves consist of an extracellular matrix (ECM), formed by proteoglycans, highly organized collagen networks and elastin fibers, and valve interstitial cells (VIC). They are surrounded by an outer layer of specialized endothelial cells (valvular endothelial cells, VEC).

Endothelial colony-forming cells (ECFCs), as a progenitor of specialized endothelial cells, are frequently used in cardiovascular research. They originate from the bone marrow and circulate in the peripheral blood, from where they can easily be isolated [2,3,4,5]. Mesenchymal stem cells (MSCs) have properties common to VICs, such as immunosuppression and antithrombogenesis [6,7,8]. Since 2007, human MSCs have been generated artificially from induced pluripotent stem cells (iPSCs) by reprogramming of non-pluripotent somatic cells [9,10]. As autologous and patient-specific cells [11], such induced MSCs (iMSCs) are particularly well suited to create functional tissue-engineered 3D heart valves.

Our group recently published the successful colonization of a 2D biodegradable poly-ε-caprolactone (PCL) nanofiber matrix with human ECFCs isolated from peripheral blood and iMSCs, differentiated from induced pluripotent stem cells (iPSCs) via mesoderm in a new fast two-phase protocol [12]. In this consecutive study, we fixed PCL scaffolds in a NiTinol stent and seeded them with human ECFCs, human MSCs, and porcine MSCs to build a prototype of a tissue-engineered 3D tri-leaflet heart valve. Colonization of the PCL scaffolds in a 3D setting and mechanical properties of the valve prototype were analyzed for the subsequent transcatheter clinical application.

## 2. Results

### 2.1. Biodegradable Poly-ε-Caprolactone (PCL) Nanofiber Scaffold

**Figure 1:**
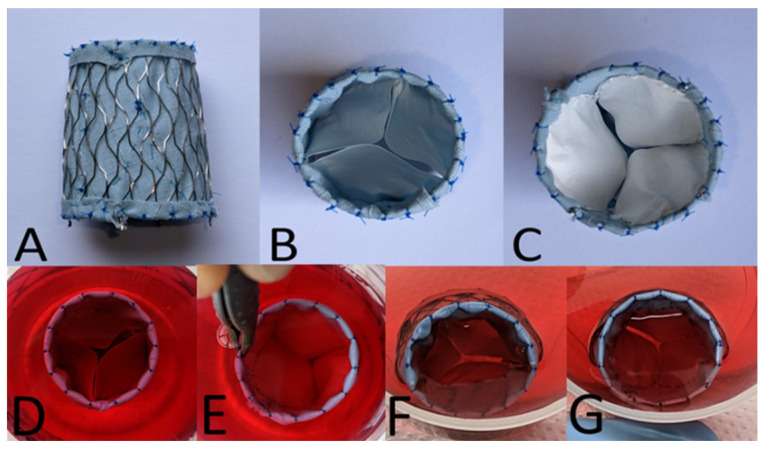
Example of a transcatheter NiTinol pulmonary heart valved stent (inner diameter 20.5 mm, outer diameter 22 mm) used in the experiments described. The lateral view (**A**) demonstrates the suturing of the inner textile cover made of polyethylene in order to prevent PVL. The top (**B**) and bottom views (**C**) illustrate the poly-ε-caprolactone (PCL) tricuspid heart valve from pulmonary and ventricular sides. D and E show pre-seeded PCL scaffolds, as a matrix with pulmonary (top, (**D**)) and ventricular ((**E**), bottom) sides. (**F**,**G**) biodegradable leaflets made of PCL nanofiber scaffolds were seeded with human induced pluripotent stem cell-derived MSCs (iMSCs) for three weeks. (**F**) shows the pulmonary (top) and G the ventricular (bottom) views.

**Figure 2:**
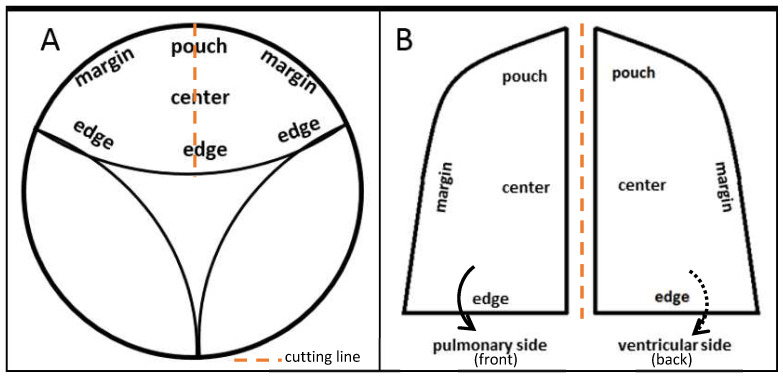
Diagrams of a valve stent (**A**) seen from the pulmonary/ventricular side (see also Figure 1B,C) and the two halves of a leaflet (**B**) highlighting the terms used to describe the different regions.

**Figure 3:**
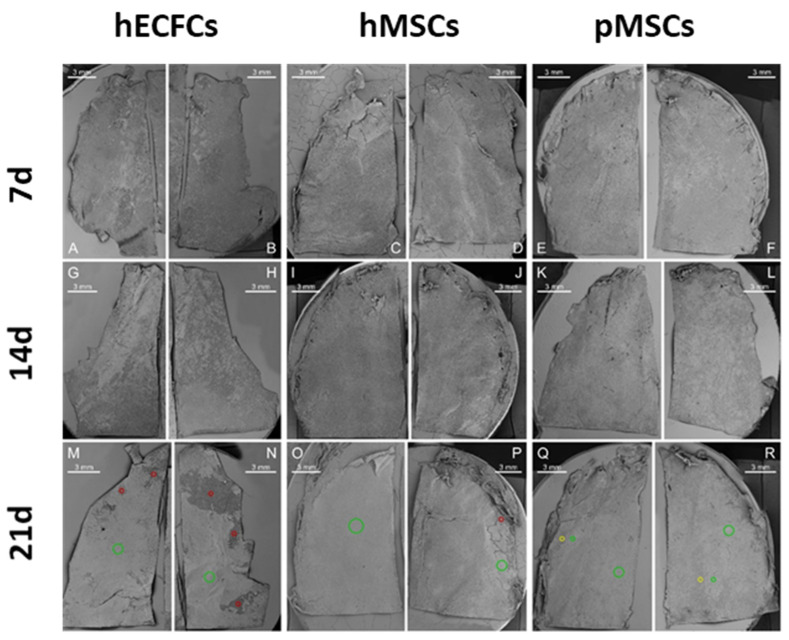
Scanning electron microscopy (SEM) images provide an overview of leaflets seeded with hECFCs (**A**,**B**,**G**,**H**,**M**,**N**), hMSCs (**C**,**D**,**I**,**J**,**O**,**P**) and pMSCs (**E**,**F**,**K**,**L**,**Q**,**R**) over time. The rows indicate results from different points in time: 1st row 7 days, 2nd row 14 days and 3rd row 21 days. Colored rings categorize vegetation cover in different areas of valve leaflets after 21 days (red = poor cell coverage, yellow = little cell coverage, green = complete cell coverage). Images were generated by combining several individual images using Affinity Photos “Panorama” function. For diagrams of leaflets and their areas see Figure 2. All images in original resolution can be found in the supplement.

**Figure 4:**
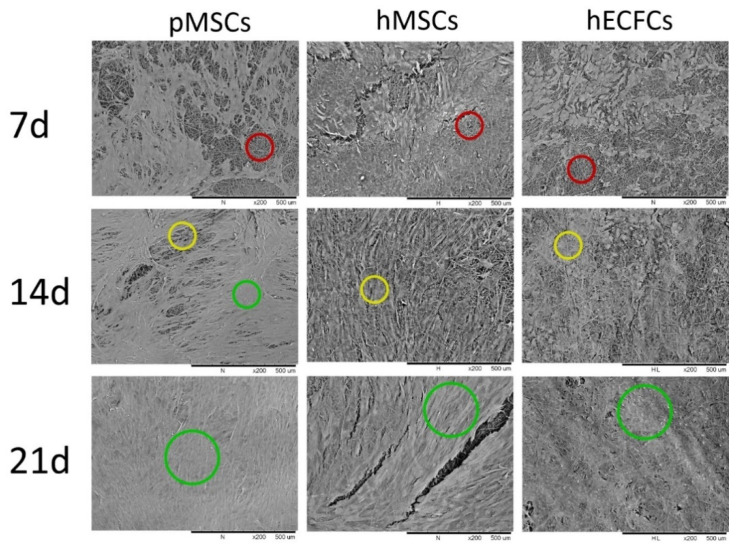
Enlarged representative scanning electron microscopy (SEM) images of the growth morphology comparing three cell lineages (pMSCs, hMSCs, and hECFCs) on uncoated PCL leaflets on days 7, 14, and 21 at 200× magnification. Colored rings categorize vegetation cover (red = poor cell coverage, yellow = moderate cell coverage, and green = complete cell coverage).

Biodegradable leaflets made of PCL nanofiber scaffolds, as a matrix (Figure 1A–G), were seeded with human induced pluripotent stem cell-derived MSCs (iMSCs) for three weeks for the generation of transcatheter tissue-engineered 3D heart valved stents (Figure 1F,G). All additional results are demonstrated in Figure 2, Figure 3 and Figure 4.

### 2.2. Growth Analysis of Different Cell Lineages

In general, all indicated lineages of cells irrespective of their species or type were able to grow on the surface of the artificial PCL leaflets. ECFCs grew largely in monolayers with a typical cobblestone pattern. Both types of MSCs with their characteristically long and thin morphology were able to grow over one another to form a gap-free cell carpet. This was especially apparent in areas where leaflets were slightly damaged due to handling and where the cell layer had peeled off the PCL matrix (see Figure 4, hECFCs and hMSCs at 21 d, pouch area).

All cell lineages were able to increase the overgrown area within the timeframe. Regardless of the cell type used, more than 90% of the leaflet area was covered with cells after three weeks. However, none of the leaflets were completely covered with cells. Interestingly, growth kinetics were different depending on the cell type. While hMSCs showed a consistent growth rate over the course of three weeks, ECFSs had almost no increase between days 7 and 14 until a growth spurt occurred between days 14 and 21.

**Figure 5:**
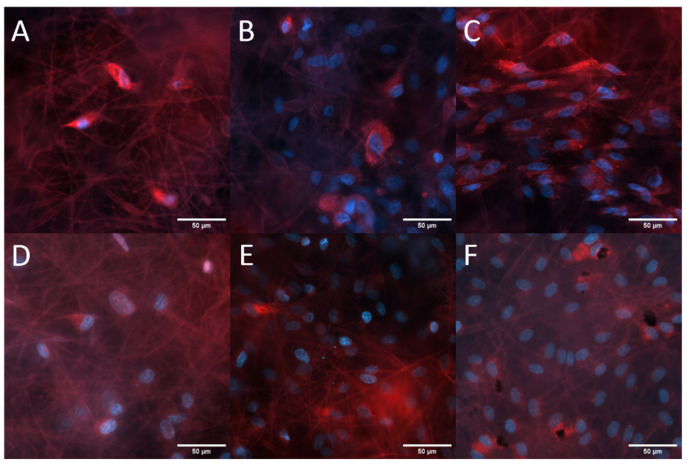
Representative fluorescence microscopy images of the morphology of the growth of MSCs and ECFCs on uncoated PCL fibers (700 nm fiber size) for up to 21 days (porcine MSCs: (**A**) 7 d, (**B**) 14 d, (**C**) 21 d, and human ECFCs: (**D**) 4 d, (**E**) 7 d, and (**F**) 14 d).

In all groups, cells grew best in the central area of the leaflets, but the attachment preferences varied in other parts (Figure 4 and Figure 5): hMSCs for example, colonized the leaflet center area and pouch faster than the leaflet edge area, while ECFSs did not seem to settle in the pouch as easily as on the edge area and center area.

Depending on the cell type, there were also differences in growth patterns between the pulmonary and ventricular sides of the leaflets. For the ECFs, there were almost no notable differences between how each side was seeded. In contrast, the hMSCs and pMSCs showed much stronger growth on the pulmonary side compared to the ventricular side.

### 2.3. Mechanical Characterization of Native Porcine Heart Valves and Colonized PCL Scaffolds

**Figure 6:**
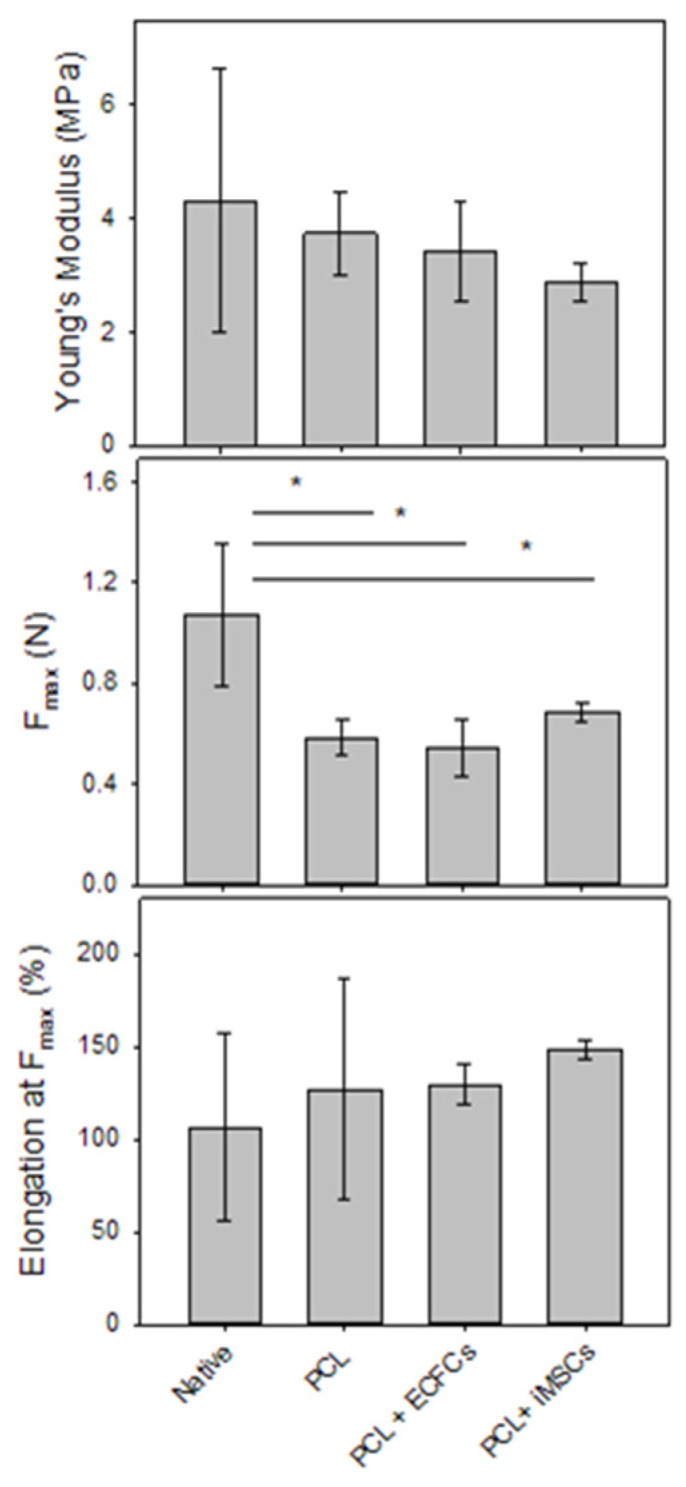
Uniaxial tensile mechanical properties. * *p* < 0.001.

To determine the mechanical properties of the colonized PCL scaffolds 14 days after seeding, they were investigated under uniaxial loading and compared to unseeded PCL scaffolds and native porcine pulmonary valves. The Young’s modulus, maximum tensile force at break (F_max_), and mean strain at break were considered in the analysis (Figure 6).

There were no significant differences in the Young’s modulus or mean strain at break between native porcine heart valves, unseeded PCL scaffolds, and PCL scaffolds colonized with human ECFCs or iMSCs (ECFCs: 3.6 ± 1 MPa and 129.4 ± 10.2% and iMSCs: 2.7 ± 0.5 MPa and 141.1 ± 5.7%, respectively) (Figure 6). In terms of these mechanical properties, PCL scaffolds were equivalent to native heart valves and did not deteriorate with cell colonization. Notably, native heart valves showed a wide standard deviation, which is was most likely due to the biological nature of the material. However, native porcine heart valves showed a significantly higher F_max_ than PCL scaffolds, which exceeds physiological levels (Figure 6).

Young’s modulus [MPa], F_max_ = maximum tensile force [N], and strain at break [%] of native porcine pulmonary heart valves, unseeded PCL scaffolds, and PCL scaffolds seeded with hECFCs or iMSCs for 14 days were tested. 

Columns are means ± standard deviation of n = 13 native valves, n = 9 PCL scaffolds, n = 10 PCL scaffolds seeded with hECFCs, and n = 5 PCL scaffolds seeded with iMSCs cells. The asterisks indicate statistically different results at a significance level of *p* < 0.05, as determined by a Kruskal–Wallis one-way analysis of variance on rank, followed by multiple means comparisons using Dunn’s method.

## 3. Discussion

### 3.1. Summary of Main Findings

The aim of this work was to produce a prototype of a 3D biodegradable and biologically active transcatheter pulmonary valve, and to investigate its biological and mechanical properties before clinical testing.

A NiTinol stent with an inner polyethylene cover served as a stent frame. For the valve leaflets, a biodegradable Poly-ε-caprolactone (PCL) scaffold was used and colonized with three different cell lines (ECFC, hMCS, and pMCS). All investigated cell lines were able to colonize more than 90 percent of the PCL scaffold within three weeks. There were differences between the cell lines in growth kinetics and the colonization of special zones, such as the free edge or outer pouch of the leaflets. Moreover, there were differences in the colonization of the pulmonary and ventricular side of the valve. Equal colonization on both sides and rapid growth on the leaflet edges was achieved with ECFCs, but colonization of the pouches was slow.

Colonization of a PCL scaffold with any cell line did not affect its mechanical properties in terms of Young’s modulus, maximal stress at break, or elongation at maximal stress. However, compared to native porcine valves, colonized PCL scaffolds did have a lower resistance to mechanical stress. Nevertheless, as already seen for PCL scaffolds, the maximal stress at break highly exceeds the physiological levels of the right ventricle and pulmonary artery, this difference might not be of relevance for clinical application. In a follow-up study, it would be interesting to investigate the differences between native valves and the tissue-engineered prototype as soon as the degradation of its PCL scaffold occurs.

### 3.2. Poly-ε-Caprolactone (PCL) for Heart Valve Tissue Engineering—Advantages and Challenges

Various biomaterials have been explored for their use in heart valve tissue engineering, including natural materials, such as decellularized valve matrices [6], and synthetic polymers, such as polyglycolic acid (PGA) and polylactic acid (PLA). Poly-ε-caprolactone (PCL) is a biodegradable polyester with favorable mechanics, which offers certain advantages over these materials. Its slow degradation rate provides extended support for cell growth and tissue remodeling, helping to create tissue-engineered heart valves that can withstand the physiological forces experienced by native valves [12]. Additionally, PCL has a lower risk of immunogenic reactions compared to decellularized valve matrices derived from allogeneic or xenogeneic sources.

In our analysis, PCL scaffolds supported cell attachment and growth (Figure 3 and Figure 4) while maintaining the structural integrity of the engineered valve construct.

The mechanical properties of the scaffold were not affected by colonization with either ECFCs or MSCs; Young’s modulus and mean strain at break were comparable between the colonized and non-colonized PCL scaffolds (Figure 6). However, native porcine heart valves were able to resist significantly higher tensile force than PCL scaffolds (F_max_ at break, Figure 6), suggesting PCL scaffolds might be prone to fail more quickly than a native valve. Optimizing PCL scaffolds to improve leaflet strength and durability will therefore be crucial for successful clinical translation.

The PCL scaffolds used in this study were fabricated using electrospinning. Electrospinning is considered the most favorable technique to produce nanofibrous structures, mimicking the native extracellular matrix and allowing optimal cell colonization, as obtained in this study [13]. The diameter of the individual fibers used was 300 nm, and the layer thickness was 100 µm. In a previous study published by our group, iMSCs colonizing 300 nm fibers showed fewer gaps and closer intercellular contact compared to those seeded on 700 nm fibers. Similar results were shown by Qu et al. in their study of a tussah silk fibroin matrix [14]. We therefore continued to use 300 nm PCL fibers for our scaffolds in this study. Higher resistance against tensile stress might be achieved with a higher fiber diameter or layer thickness. Moreover, we relied on randomly orientated fibers instead of aligned fibers, as their larger pore size facilitated cell adhesion, as shown in a study conducted in our laboratory. Other studies suggest faster migration of cells in aligned fiber scaffolds [14]. Further research on fiber diameter and layer thickness as well as cell adhesion on aligned fiber scaffolds is therefore required to optimize PCL scaffolds to achieve the best equivalence to native valves.

### 3.3. Generation of Transcatheter 3D Tissue-Engineered Tricuspid Valved Stents

A major limitation of current valve prostheses is the absence of cells required for active repair and remodeling of the valve matrix. Stem cells, with their capacity for self-renewal and differentiation, have emerged as a promising cell source for heart valve tissue engineering. Their ability to generate various cell types, including endothelial cells and valve interstitial cells, allows for the formation of functional valve structures with an appropriate extracellular matrix composition [7,15]. Furthermore, stem cells can contribute to tissue remodeling and repair, promoting the integration of tissue-engineered valves within the native tissue environment.

#### 3.3.1. Different Sources of Stem Cells, Advantages and Challenges for Heart Valve Tissue Engineering

Various stem cell sources have been explored for heart valve tissue engineering, including adult stem cells, such as bone marrow-derived mesenchymal stem cells (MSCs) [8] and endothelial progenitor cells (EPCs) [4]. Recently, induced pluripotent stem cells (iPSCs) have attracted interest due to their ability to differentiate into various cell types and their potential for patient-specific customization [9,10]. iPSCs can be generated by reprogramming adult somatic cells, reducing the ethical concerns and immunogenicity associated with other stem cell sources [11].

Stem cells offer several advantages for heart valve tissue engineering, including their potential to differentiate into various cell types, contribute to tissue remodeling, and reduce immunogenicity when using patient-specific iPSCs. However, challenges remain in optimizing the differentiation protocols, ensuring the safety and efficacy of stem cell-derived valve constructs, and understanding the long-term outcomes of stem cell-based valve replacements.

#### 3.3.2. Comparisons with Other Studies Using Stem Cells for Heart Valve Tissue Engineering

This study utilized human-induced pluripotent stem cell-derived MSCs (iMSCs) and ECFCs to generate transcatheter tissue-engineered 3D heart valved stents using biodegradable PCL nanofiber scaffolds. This approach is supported by other studies that have investigated various stem cell sources and scaffold materials for heart valve tissue engineering.

For example, a study by Hashi et al. [8] demonstrated the antithrombogenic properties of bone marrow-derived MSCs when seeded onto nanofibrous scaffolds for vascular graft applications. Similarly, a study by Latif et al. [7] characterized the signaling and structural molecules produced by human valve interstitial cells and MSCs, highlighting their potential for valve tissue engineering.

The use of blood-derived ECFCs and iMSCs is particularly appealing because they can both be obtained in a non-invasive manner, greatly expanded in vitro, allowing for the colonization of PCL scaffolds with autologous cells to enable the biological integration of a living heart valve [12]. Besides, these cells offer potential advantages in terms of patient-specific customization and reduced immunogenicity compared to allogeneic or xenogeneic cell sources.

In summary, in this study, we analyzed the growth of ECFCs, and human and porcine MSCs on a PCL nanofiber matrix in 3D transcatheter pulmonary heart valved stents; cell adhesion, proliferation, and distribution on uncoated PCL fibers were especially closely examined using SEM after cellular growth on 3D heart valve leaflets made of PCL fibers (Figure 3 and Figure 4). The ECFCs adhered and proliferated on PCL fibers of the 3D valved stent and formed a gap-free cell layer on day 21. Small gaps in the cell cover were associated with imperfections in sample processing. Even though the highest cell number was observed after three weeks of cell culturing, cells were evenly distributed and more elongated than earlier in the study.

The same was true for the human and porcine iMSCs as they adhered and proliferated on uncoated PCL fibers and formed a nearly closed cell layer. They appeared evenly distributed and elongated after three weeks of cell culturing: more than 90% of the heart leaflets were completely covered with cells in nearly all leaflet areas regardless of which cell type was used. Additionally, the use of iMSCs as a cell source offers potential advantages in terms of patient-specific customization and reduced immunogenicity compared to allogeneic or xenogeneic cell sources.

## 4. Materials and Methods

### 4.1. Ethics Statement

Human induced pluripotent stem cells (iPSC) generation and biobanking was approved by the Ethics Committee (21 January 2011 and 10 September 2015) of the University Medical Center at Göttingen, Germany. The collection of peripheral blood for ECFC isolation was approved by the local Ethical Committee (D464/2016). All patients provided written informed consent before recruitment after receiving a full explanation of the study. The study procedures were approved by the Ethics Committee of the Medical Faculty of Medicine at Kiel University, Germany (approval number: D522/2016).

### 4.2. Endothelial Colony Forming Cells (ECFC) Isolation and Culture

Human ECFCs were isolated from peripheral blood according to the previously published protocol with minor modifications [12].

### 4.3. Characterization of ECFCs

Cells in passage 5 were characterized using the Matrigel tube formation assay as previously described [12].

Tube formation was examined every two hours using phase contrast microscopy (Zeiss, Jena, Germany). For flow cytometry, cells were washed with PBS and resuspended in a PBS staining solution with 2% foetal bovine serum (FBS). Thereafter, cell samples were separately labelled on ice within an optimal dilution of conjugated monoclonal antibodies (BioLegend, San Diego, CA, USA), as described before [12].

### 4.4. Characterization of Mesenchymal Stem Cells (iMSCs)

Flow cytometric analysis, as described above for ECFCs, was used to detect the cell surface antigen profile of iMSCs (generated from two human iPS cell lines, as described before) [12]. The following monoclonal antibodies (BioLegend) were used: allophycocyanin (APC), mouse anti-human CD34, FITC mouse anti-human CD44, APC/Cy7 mouse anti-human CD45, PE/Cy7 mouse anti-human CD90, FITC mouse anti-human CD146, and PE mouse anti-human CD166 [12].

### 4.5. Porcine Mesenchymal Stem Cells (pMSCs)

Porcine MSCs were commercially purchased from Cell Biologics Inc. (Chicago, IL, USA) and expanded in MSC growth medium consisting of Dulbecco’s modified Eagle medium–high glucose (DMEM-HG; Gibco), 10% defined FBS (Gibco), and 1% penicillin–streptomycin (Gibco) on uncoated culture dishes and kept at 5% CO_2_ and at 37 °C. For routine expansion, cells were plated at 5 × 10^4^ cells cm^2^ with the culture medium changed every 3–4 days.

### 4.6. Electrospun PCL Scaffolds

Randomly oriented biodegradable electrospun PCL scaffolds, purchased from Nanofiber Solutions (Dublin, OH, USA), with a fiber size of 300 nm in diameter and a layer thickness of 100 µm were used for seeding experiments. The random orientation of the fibers mimics the 3D nanofibrous extracellular matrix found throughout the body. The increased surface of the scaffold as well as its pores facilitate the adhesion of higher cell numbers.

### 4.7. PCL Heart Valved Stents

Nickel–titanium (Nitinol) pulmonary stents were produced by Optimed GmbH (Ettlingen, Germany). The stent frame was covered with polyesther fabric to avoid potential leakage. Leaflets were cut from PCL scaffold with a fiber diameter of 300 nm using stencils and sutured into the stent using a polypropylene suture (Prolene, Johnson & Johnson, New Brunswick, NJ, USA). All leaflets within the stents were marked with a knot on the ventricular side to make later identification easier. Stents were sterilized in 0.15% peracetic acid (AppliChem, Darmstadt, Germany) for 2 h and washed 3 × 15 min with PBS. Prior to the start of the experiments, the valved stents were incubated in the culture medium overnight at 5% CO_2_ and 37 °C.

### 4.8. Dynamic Cell Seeding onto PCL Scaffolds

The seeding process was conducted with a cell seeder (Aptus Bioreactors, Clemson, SA, USA) in a humidified atmosphere at 37 °C and 5% CO_2_ (bioreactor) and performed for pMSCs, hMSCs, and hECFCs in a similar manner. On Day 1, cells were extracted from culture flasks (approximately 6 million cells for pMSCs, 8.7 million cells for hMSCs, and 12.6 million cells for hECFCs) and dripped onto the surface of the closed valve (pulmonary side). Afterwards, each seeding chamber/bioreactor was filled up to 70 mL with preheated culture medium (37 °C). Prior to starting the motion program of the seeder (speed: 4 rpm at a 30° angle to each side), the valved stent was left to rest in the incubator for 3 h, to facilitate cell adhesion onto the leaflet surface. On Day 3, the culture medium was exchanged and additional cells (approximately 5 million cells for pMSCs, 4.7 million cells for hMSCs, and 4.46 million cells for ECFCs), were dripped onto the leaflets of the horizontally placed stent. The leaflets of the pulmonary valved stents were rotated regularly during the process to ensure equal distribution of all cells onto each of the three leaflets.

Subsequently, the same program for the seeder (speed: 4 rpm at a 30° angle to each side) was used for the next 19 days and the culture medium was replaced two (for both types of MSCs) or three times (for hECFCs) per week. On Days 7, 14, and 21 one of the leaflets was cut out using a scalpel. In preparation for further analysis, the leaflets were washed three times in PBS and fixed in phosphate buffered formaldehyde (ROTI*Histofix 10%, Roth) for 2 h at 4 °C.

### 4.9. Fluorescent Staining

Cells were washed and blocked with medium supplemented with 10% goat serum (PAN Biotech, Aidenbach, Germany) for 20 min at 37 °C. Afterwards, they were stained with Hoechst 33342 and CellTracker Red CMTPX (both Thermo Fisher Scientific, Waltham, MA, USA) according to the manufacturer’s instructions. The staining solution was incubated for 30 min at 37 °C. Then, PCL was analyzed using an Axio Observer fluorescence microscope (Carl Zeiss, Jena, Germany) after washing with PBS [12].

### 4.10. Biomechanical Examination

The biomechanical characteristics of native porcine leaflets and 5 × 15 mm strips of PCL tissue (unseeded, but soaked in nutrient medium, and seeded for 14 days), were analyzed using uniaxial tensile testing. The strips from the seeded PCL scaffolds were taken from the central region of the leaflet. Tensile tests were performed using a universal testing machine (ZwickRoell Z0.5, Ulm, Germany) with a 0.5 kN load cell with a sensitivity of 2 mV/V. Samples were stretched at 5.0 mm/min until complete rupture. The maximum tensile force (F_max_) and elongation at break were recorded [12]. To determine the leaflet’s resistance to external forces, the Young’s modulus was calculated, dividing F_max_ at break per cross-sectional area by the elongation at break [12].

### 4.11. Scanning Electron Microscopic (SEM) Analysis

Sample preparation was carried out as described before [12]. All images were obtained using a Hitachi TM3000 (Hitachi High-technologies Corp., Tokyo, Japan) at 15 kV acceleration voltage at different magnifications (50×, 200×, and 1000×). Images were post-processed using Affinity Photo, Affinity Designer, and Affinity Publisher (Serif Ltd., Bridgford, UK). Composite images of leaflets were generated by combining several individual images using Affinity Photos “Panorama” function.

### 4.12. Statistical Analysis

Sigmaplot 13 (Systat Software GmbH, Erkrath, Germany) was used for statistical analysis. Kruskal–Wallis one-way analysis of variance was used to compare groups, and where significant differences were found, the Dunn’s method was used for the comparison of means.

## 5. Conclusions

This study utilized colonized PCL nanofiber scaffolds in a NiTinol stent frame to form a biodegradable and biologically active transcatheter pulmonary valved stent. This new approach combined the advantages of synthetic biodegradable materials with patient-specific cell sources, offering potential improvements in valve performance and long-term outcomes for right ventricular outflow obstruction.

## Data Availability

Data contained within the article were revised according to the journal rules.

## References

[1] Ciubotaru A., Cebotari S., Tudorache I., Beckmann E., Hilfiker A., Haverich A. (2013). Biological heart valves. Biomed. Tech. Eng..

[2] Pelosi E., Castelli G., Testa U. (2014). Endothelial progenitors. Blood Cells Mol. Dis..

[3] Yoder M.C. (2012). Human Endothelial Progenitor Cells. Cold Spring Harb. Perspect. Med..

[4] Tasev D., Koolwijk P., van Hinsbergh V.W.M. (2016). Therapeutic Potential of Human-Derived Endothelial Colony-Forming Cells in Animal Models. Tissue Eng. Part B Rev..

[5] Glynn J.J., Hinds M.T. (2014). Endothelial Outgrowth Cells: Function and Performance in Vascular Grafts. Tissue Eng. Part B Rev..

[6] Iop L., Renier V., Naso F., Piccoli M., Bonetti A., Gandaglia A., Pozzobon M., Paolin A., Ortolani F., Marchini M. (2009). The influence of heart valve leaflet matrix characteristics on the interaction between human mesenchymal stem cells and decellularized scaffolds. Biomaterials.

[7] Latif N., Sarathchandra P., Thomas P.S., Antoniw J., Batten P., Chester A.H., Taylor P.M., Yacoub M.H. (2007). Characterization of structural and signaling molecules by human valve interstitial cells and comparison to human mesenchymal stem cells. J. Heart Valve Dis..

[8] Hashi C.K., Zhu Y., Yang G.-Y., Young W.L., Hsiao B.S., Wang K., Chu B., Li S. (2007). Antithrombogenic property of bone marrow mesenchymal stem cells in nanofibrous vascular grafts. Proc. Natl. Acad. Sci. USA.

[9] Okita K., Ichisaka T., Yamanaka S. (2007). Generation of germline-competent induced pluripotent stem cells. Nature.

[10] Yu J., Vodyanik M.A., Smuga-Otto K., Antosiewicz-Bourget J., Frane J.L., Tian S., Nie J., Jonsdottir G.A., Ruotti V., Stewart R. (2007). Induced Pluripotent Stem Cell Lines Derived from Human Somatic Cells. Science.

[11] Sackett S.D., Brown M.E., Tremmel D.M., Ellis T., Burlingham W.J., Odorico J.S. (2016). Modulation of human allogeneic and syngeneic pluripotent stem cells and immunological implications for transplantation. Transplant. Rev..

[12] Lutter G., Puehler T., Cyganek L., Seiler J., Rogler A., Herberth T., Knueppel P., Gorb S.N., Sathananthan J., Sellers S. (2022). Biodegradable Poly-ε-Caprolactone Scaffolds with ECFCs and iMSCs for Tissue-Engineered Heart Valves. Int. J. Mol. Sci..

[13] Dwivedi R., Kumar S., Pandey R., Mahajan A., Nandana D., Katti D.S., Mehrotra D. (2020). Polycaprolactone as biomaterial for bone scaffolds: Review of literature. J. Oral Biol. Craniofacial Res..

[14] Qu J., Zhou D., Xu X., Zhang F., He L., Ye R., Zhu Z., Zuo B., Zhang H. (2012). Optimization of electrospun TSF nanofiber alignment and diameter to promote growth and migration of mesenchymal stem cells. Appl. Surf. Sci..

[15] Kutikhin A.G., Tupikin A.E., Matveeva V.G., Shishkova D.K., Antonova L.V., Kabilov M.R., Velikanova E.A. (2020). Human Peripheral Blood-Derived Endothelial Colony-Forming Cells Are Highly Similar to Mature Vascular Endothelial Cells yet Demonstrate a Transitional Transcriptomic Signature. Cells.

